# Dynamics and disorder: on the stability of pyrazinamide polymorphs

**DOI:** 10.1107/S2052520622004577

**Published:** 2022-06-01

**Authors:** Anna Agnieszka Hoser, Toms Rekis, Anders Østergaard Madsen

**Affiliations:** a Biological and Chemical Research Centre, Faculty of Chemistry, University of Warsaw, Żwirki i Wigury 101, Warszawa, 02-089, Poland; b Department of Pharmacy, University of Copenhagen, Universitetsparken 2, Copenhagen, 2100, Denmark

**Keywords:** polymorphism, enantiotropism, normal-mode refinement, periodic density functional theory, quantum crystallography

## Abstract

This article focuses on the structure and relative stability of four pyrazinamide polymorphs. New single crystal X-ray diffraction data collected for all forms at 10 K and 122 K are presented. By combining periodic *ab* *initio* DFT calculations with normal-mode refinement against X-ray diffraction data, both enthalpic and entropic contributions to the free energy of all polymorphs are calculated. On the basis of the estimated free energies, the stability order of the polymorphs as a function of temperature and the corresponding solid state phase transition temperatures are anticipated. It can be concluded that the α and γ forms have higher vibrational entropy than that of the β and δ forms and therefore they are significantly more stabilized at higher temperatures. Due to the entropy which arises from the disorder in γ form, it overcomes form α and is the most stable form at temperatures above ∼500 K. Our findings are in qualitative agreement with the experimental calorimetry results.

## Abstract 2

The enantiotropic relationship between the four polymorphs of pyrazinamide is analyzed by means of accurate X-ray diffraction measurements, normal-mode refinement and periodic DFT calculations.

## 1. Introduction

The understanding of crystalline phase thermal stability is of paramount importance for drug systems, because it influences both the shelf-life and the bioavailability of solid state dosage forms. The stability of the crystalline phase is a matter of thermodynamic and kinetic factors. Thermodynamically, the crystal must be energetically more stable than any other phase – be that the liquid, gas or other solid-state forms. Meanwhile, the lack of thermodynamic stability may not lead to a transformation, if there is no feasible pathway from one form to another – in other words, kinetics has the final word in determining the fate of our crystalline phase.

Even without considering such complicating kinetic factors, the matter can become rather complex. In a polymorphic system, only one structure is the thermodynamically stable form at any given temperature and pressure. Enantiotropic polymorphism – where the thermodynamic stability of polymorphs changes order as a function of temperature – can be explained by differences in entropy as well as enthalpy. Entropic differences can arise due to both disorder and thermal motion. In this work, we will investigate such an enantiotropic system – pyrazinamide – and will try to elucidate the different contributions that lead to its enantiotropic behavior by means of a combination of computations (periodic density functional theory (DFT) calculations) and X-ray diffraction measurements.

From a pharmaceutical perspective, pyrazinamide is a very well known anti-tuberculosis drug listed by the World Health Organization on the Model List of Essential Medicines. It also exhibits strong antipyretic, fibrinolytic and antibacterial properties.

**Figure 1:**
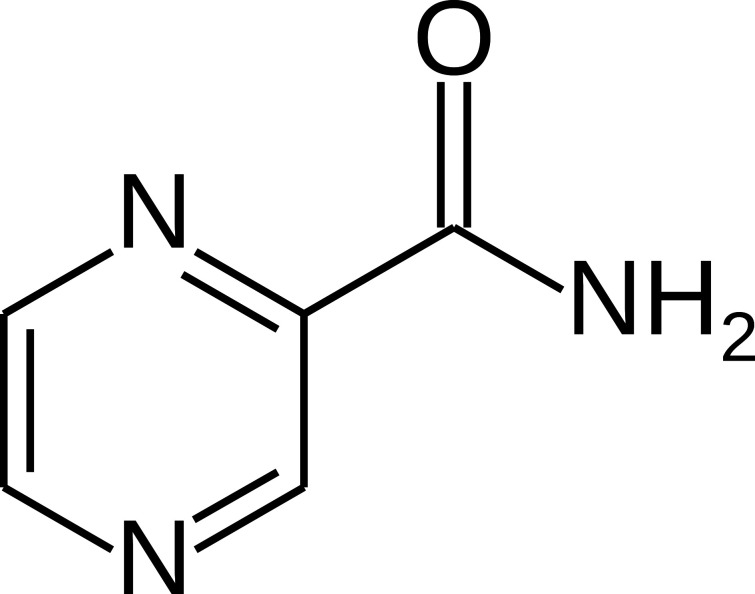
Molecular structure of pyrazinamide.

**Figure 2:**
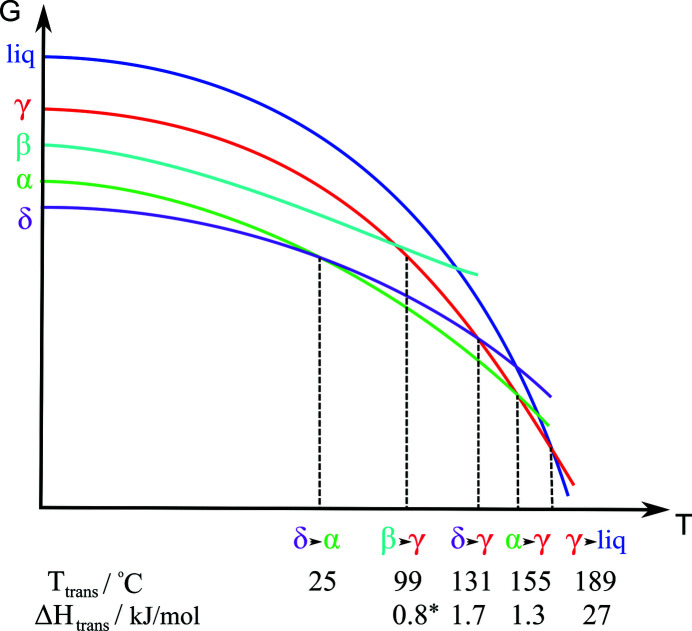
Sketch of a *G*–*T* diagram which is in accordance with the observed phase transformations. The implied phase transformations, the transformation temperatures and the enthalpies of transformation derived from DSC measurements are given below the ordinate. The diagram is based on information and similar diagrams in previous work (Castro *et al.*, 2010 ▸; Cherukuvada *et al.*, 2010 ▸). The (*) implies that the transition enthalpy of the β to γ transition is not well determined, because the β sample was contaminated by γ-form crystals.

Despite its very simple molecular structure (Fig. 1 ▸), pyrazinamide has a complex solid form landscape; to this day four polymorphs have been found, and their structures (crystallographic data in Table 1 ▸) are well known (Takaki *et al.*, 1960 ▸; Tiwari *et al.*, 1982 ▸; Rø & Sørum, 1972 ▸; Cherukuvada *et al.*, 2010 ▸; Castro *et al.*, 2010 ▸; Wahlberg *et al.*, 2014 ▸). The forms can be obtained by standard crystallization techniques such as slow evaporation of solvents or sublimation. In many cases, the polymorphs crystallize concomitantly. Based on a plethora of experimental evidence, including calorimetric measurements, hot-stage microscopy observations, spectroscopic measurements, grinding experiments, and long-term storage and powder X-ray diffraction (PXRD) diagrams, the relative stability of the polymorphs as a function of temperature has been proposed (Castro *et al.*, 2010 ▸; Cherukuvada *et al.*, 2010 ▸). Furthermore, tentative diagrams of the free energy versus temperature for the four forms and the liquid state is available (see Fig. 2 ▸).

### 1.1. Description of stabilities

The solid-state phase transformations can be observed using differential scanning calorimetry (DSC). DSC provides phase transition temperatures and enthalpies, although these data have to be taken *cum grano salis*, since they are influenced by kinetics and by the phase purity of the sample. In particular, a recent study points to the difficulties in assessing the exact temperature of the α to γ transition (Li *et al.*, 2020 ▸) because the transition temperature depends on the DSC heating rate. Based on the phase transformations observed during storage of crystals at a range of temperatures, Li *et al.* propose a transformation temperature of 392 (1) K. The difference in solid state enthalpies between α and γ seems to be temperature independent and amounts to 13.2 (6) J g^−1^ corresponding to 1.6 (1) kJ mol^−1^, with the α form being enthalpically the most stable.

Based on the observed phase transformations from DSC, hot-stage microscopy, Raman and PXRD measurements, it appears that at low temperatures, the γ form is the least stable – yet at higher temperatures all other forms – α, β and δ – transform to γ before reaching the melting point (Castro *et al.*, 2010 ▸, Cherukuvada *et al.*, 2010 ▸). Additionally, already at room temperature the δ form can transform into the α form. The free energy versus temperature diagram in Fig. 2 ▸ agrees with these observations. However, several other *G*–*T* diagrams could be sketched: in particular, it is difficult to draw any conclusions of the relative stabilities at 0 K for the β form in relation to the α and δ forms. Although the transformation to γ appears to occur at a lower temperature than for α and δ, the β form could be the most stable at 0 K if the *G*–*T* line for β is less curved than for the other forms. This would imply that the *G*–*T* curve of β would cross the curves of α and δ, implying possible phase transformations – which, however, could be kinetically hindered.

To get an understanding of the different contributions giving rise to a temperature dependence of the relative thermodynamic stabilities of the four polymorphs, it is necessary to obtain an insight into the enthalpic as well as entropic contributions to the free energy, *i.e.* to calculate *G* = *H* − *TS*, where *H* is the enthalpy, *T* is the temperature and *S* is the entropy. In this work, we use a combination of periodic DFT calculations and X-ray diffraction measurements to obtain an estimate of the free energy *G* at different temperatures, as outlined below.

### 1.2. Entropic contribution to thermodynamic stabilities

At absolute zero, a perfect crystal has zero entropy. However, disorder in the crystalline phase may give a contribution to the entropy even at *T* = 0 K.

It is well known that the γ form of pyrazinamide is disordered (Cherukuvada *et al.*, 2010 ▸). The crystal structure shows that 15% of the molecules are rotated (*vide infra*). On one hand, this disorder gives rise to a small decrease in the enthalpic stability of the crystal, while on the other hand stabilizes the crystal entropically.

Whereas the disorder of the γ form is readily visible in the electron density derived from single-crystal diffraction experiments, we have found it interesting to investigate whether the other polymorphs are disordered in any way. Therefore, we have conducted measurements at 10 K. At this temperature, disorder that is not visible at higher temperatures – *i.e.* hiding as a contribution to the displacement ellipsoids – can be revealed. At 10 K the intermolecular vibrations are significantly reduced. The disorder will manifest itself as splitting of atomic positions, or as significantly larger ellipsoids than what can be expected to arise from intramolecular motion.

Apart from the disorder, the most substantial contribution to the entropy in the solid state is from the thermal vibrations in the crystal. Vibrational contributions to free energy are demanding to obtain from theoretical calculations for molecular crystals due to high computational costs, and because the accuracy of the calculated low-frequency phonons may be low. Thus, for the pyrazinamide polymorphs we apply a recently developed quantum crystallography method (Genoni *et al.*, 2018 ▸), coined Dynamic Quantum Crystallography, of estimating vibrational entropy and enthalpy from frequencies refined against single crystal X-ray diffraction data (Hoser & Madsen, 2016 ▸; 2017 ▸). We term this refinement a *normal-mode refinement* (NoMoRe), and in recent years we have explained, validated, and applied this approach to various systems to estimate thermodynamic properties (Hoser *et al.*, 2021 ▸; Sovago *et al.*, 2020 ▸; Kofoed *et al.*, 2019 ▸).

### 1.3. Enthalpic contribution to thermodynamic stability

While the NoMoRe procedure ensures an estimate of the vibrational entropy and enthalpy, the enthalpic differences due to inter- and intramolecular conformations of the molecules are not considered. In recent years, periodic DFT calculations including an empirical estimate of dispersion interactions have been shown to be able to give reasonable estimates of differences in crystal polymorph enthalpies and has been an important reason for the success of crystal structure prediction (Reilly *et al.*, 2016 ▸). However, in our previous work (Wahlberg *et al.*, 2014 ▸) we have explored how differences in the crystal geometries of pyrazinamide can lead to rather different DFT-estimated stabilities of the pyrazinamide polymorphs.

In the present contribution, we update that work using new methodology, in particular with respect to the empirical estimates of the dispersion forces. Next, we estimate contributions to the Gibbs free energies that arise from vibrations and disorder and we predict the ordering of stability of polymorphic forms at different temperatures.

## 2. Materials and methods

### 2.1. Single-crystal X-ray diffraction

Crystalline pyrazinamide, purchased from Sigma, was used without further purification. The α, β and δ polymorphs were crystallized from the saturated solution by slow evaporation at room temperature. The respective solvents were water, chloro­form and di­chloro­methane, in accordance with the work of Castro *et al.* (2010 ▸). Crystals of the α form were slowly heated in an oven to 160 °C and in what appears to be a single-crystal to single-crystal transformation turned to the γ form.

The diffraction measurements were performed using Bruker/Nonius in-house X-ray diffractometers, and the temperature was controlled by use of nitro­gen and helium open gas-flow systems (from Cryo Industries of America). All data were collected using molybdenum radiation and with area detectors. For each polymorph, data at 10 K and 122 K were collected. The 10 K data were limited to a resolution of maximum sin(θ)/λ = 0.8 Å^−1^, whereas the 122 K data were obtained to at least sin(θ)/λ = 1.0 Å^−1^. The data were integrated and reduced using the *SAINT* program.

For the deposited structures, the software *JANA2006* (Petříček *et al.*, 2014 ▸) was used for structure solution and refinement. Anisotropic displacement parameters were refined for non-hydrogen atoms. The hydrogen atoms were fixed at calculated positions and refined using the riding model. For the γ polymorph, a rigid-body approach was used for the minor disorder component.

The *SHELXL* program (Sheldrick, 2015 ▸) was used for block-refinement as part of the NoMoRe refinement.

An overview of the crystallographic information can be found in Table 1 ▸.

### 2.2. Theoretical calculations

Periodic *ab*-*initio* density functional theory (DFT) calculations were performed for the selected systems using the *CRYSTAL17* program (Dovesi *et al.*, 2017 ▸).

**Electronic energy calculations**. Electronic energies were calculated with two different functionals B3LYP (Lee *et al.*, 1988 ▸) and PBE0 (Adamo & Barone, 1999 ▸) in combination with an D3 empirical dispersion (Grimme *et al.*, 2010 ▸) energy correction and with a large pVTZP (Schäfer *et al.*, 1994 ▸) basis set with polarization functions. The truncation parameters, TOLINTEG, were set to 7 7 7 7 25 and the shrinking factors in the reciprocal space, SHRINK, were set to 8 and 8.

Moreover, we tried two approaches – full geometry optimization and optimization of atom coordinates only, with cell parameters fixed at those from the 10 K measurements.

**Phonon calculations** were performed in *CRYSTAL17* to provide an ansatz for the Normal Mode Refinements (NoMoRe). The standard 6-31G(d,p) basis set was applied. It has been used extensively and gives reasonable relative stabilities of related systems; it has also been used for the estimation of vibrational frequencies. We optimized the geometry (only the coordinates, starting from the experimental geometry). Subsequently, we calculated the normal modes and their frequencies at the Γ point of the Brillouin zone. The convergence criteria for geometry optimization were set to the default for frequency calculations using the PREOPTGEOM keyword. As we optimized only the coordinates, the frequencies were calculated using the cell parameters from the 122 K XRD measurements. Input for *CRYSTAL17* frequency calculations can be easily created using our cif2crystal routine (http://shade.ki.ku.dk/docs/cif2crystal.html).

### 2.3. The normal-mode refinements (NoMoRe)

We used the nomore.chem.uw.edu.pl web server (a version of the NoMoRe Python program) to conduct refinements against pyrazinamide polymorph X-ray data from 122 K. For α, β and δ we submitted normal-mode vectors and their frequencies obtained from theoretical calculations, structure factor files and input files for *SHELXL* with a model of the given structure. For each polymorphic form we refined six frequencies, corresponding to the low-energy acoustic and optical phonons, which lead us to reasonable models (with quite low agreement indices (*wR* ^2^) and anisotropic displacement parameters (ADPs) similar to those from routine *SHELXL* refinement, see supporting information). The NoMoRe python program was adapted to refine the disordered γ polymorph. During these refinements, the parameters describing the minor disorder component were kept constant. The electron density related to the minor disorder component does not overlap with the density of the major component, and thereby the displacement parameters of the major component appear unaffected by the disorder.

### 2.4. Evaluation of thermodynamic functions

In the case of all polymorphic forms we used known thermodynamic equations, which we already applied for polymorphs of di­methyl-3,6-di­chloro-2,5-di­hydroxy­terephthalate (Kofoed *et al.*, 2019 ▸). For each polymorph the free energy (G) is calculated as it is shown in the following equations:

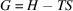

where *H* is enthalpy, *T* is temperature in K and *S* is entropy. The enthalpic part can be divided into *U*, crystal packing energy, and a 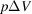 , pressure × volume, term which take into account the expansion of the unit cell with temperature

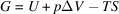

(where the *p*Δ*V* term is calculated at a pressure of 1 atm), whereas

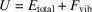


*E*_total_ is an electronic energy which is accessible from DFT calculations and the contribution from vibrational energy, *F*_vib_ is

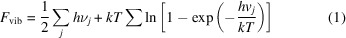

Where the summation in equation (1) runs over frequencies ν, *h* is Planck’s constant and *k* is the Boltzmann constant. The first part of the equation is the zero-point energy (ZPE), while the second part is the contribution to enthalpy (Hvib) coming from normal-mode vibrations. In a similar fashion, the vibrational entropy can be obtained by summation over all normal-mode vibrations:

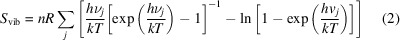

In the case of NoMoRe all the frequencies obtained from the calculations in *CRYSTAL17* (and for which a subset was subsequently refined against the diffraction data) were used in order to obtain full vibrational contributions to free energy.

For the γ form, we additionally took into account the entropy arising from disorder, as outlined below.

### 2.5. Entropy of mixing for γ polymorphic form

Disorder can stabilize the γ pyrazinamide structure by increasing the entropy. We estimate these entropy contributions by calculating the *entropy of mixing*.

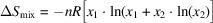

where *x*_1_ and *x*_2_ are the experimentally observed occupancies (0.15 and 0.85).

## 3. Results and discussion

### 3.1. Structures and intermolecular interaction description

The structures of the pyrazinamide polymorphs have already been described and are well known. The data for all pyrazinamide polymorphs obtained in this study at 122 K are of a high-resolution [sin(θ)/λ > 1.0 Å^−1^], giving more confidence in the atomic positions and the description of disorder than previous studies. Previously, a comparable resolution data of sin(θ)/λ > 1.0 Å^−1^ have only been reported for α and β polymorphs (Rajalakshmi *et al.*, 2014 ▸; Jarzembska *et al.*, 2014 ▸). The data obtained at 10 K are of a slightly lower resolution [sin(θ)/λ ≃ 0.8 Å^−1^], simply due to experimental limitations where the cost of helium sets a limit to the experiment time. However, the displacement parameters of the α, β and δ polymorphs derived from the 10 K data indicates that there is no disorder in these systems. The γ form is thereby the only form where a more advanced model is needed for the calculation of the enthalpic contribution to the free energy via periodic DFT calculations.

**Figure 3:**
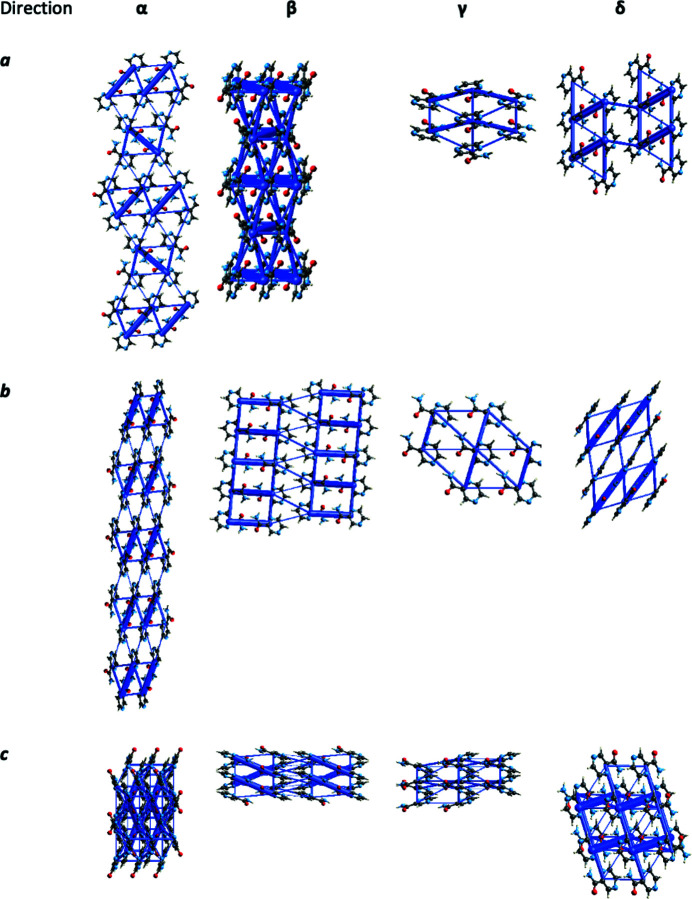
Energy frameworks generated for pyrazinamide polymorphs [*CrystalExplorer* (Spackman *et al.*, 2021 ▸), B3LYP level of theory].

Comparing energy frameworks (Turner *et al.*, 2015 ▸, Spackman *et al.*, 2021 ▸) generated for pyrazinamide polymorphs (Fig. 3 ▸) we immediately see that in the α, β and δ polymorphs dimers are formed by pyrazinamide molecules via the hydrogen bonding between amide groups. For those structures, this is the strongest interaction. Such dimers are stabilized by other weaker - mostly dispersive - interactions. The γ form is a remarkable exception – here, neighboring pyrazinamide molecules form chains via hydrogen bonding interactions of amide groups with nitro­gen from the aromatic ring. Furthermore, such chains are stabilized by dispersive interactions (see supporting materials). Therefore, proper dispersion corrections are crucial for theoretical calculations for this system.

**Figure 4:**
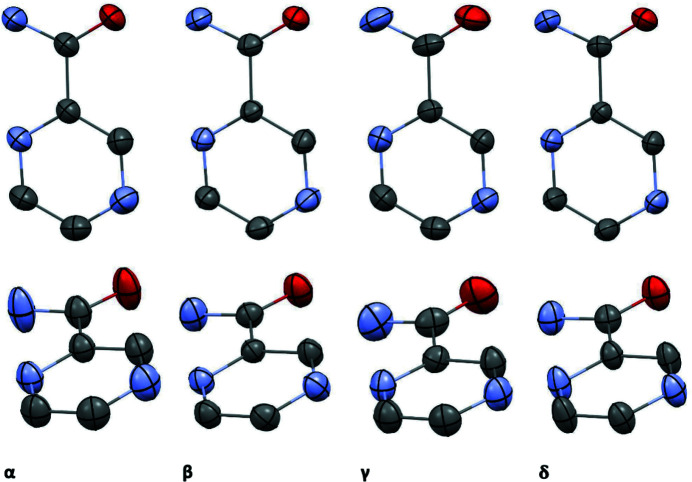
Visual comparison of non-hydrogen ADPs from 122 K. Ellipsoids are depicted at the 90% probability level.

Comparison of anisotropic displacement parameters (ADPs) reveals that ADPs for the α and γ forms are significantly larger than those for the other polymorphic forms (Fig. 4 ▸). In the case of the α form this observation can be rationalized by comparison of unit-cell volumes and densities for different polymorphic forms – the α form has significantly higher cell volume and lower density than the other polymorphic forms – thus, the atoms have more space to vibrate and in consequence the ADPs are larger.

Large ADPs for the α and γ forms are a hint that the vibrational entropy can give important contributions to the relative stability of polymorphic systems. However, the system with the largest ADPs does not always have the largest entropy, because the intramolecular vibrations can play a significant role, as we have demonstrated previously (Kofoed *et al.*, 2019 ▸).

### 3.2. On the disorder of γ polymorph

**Figure 5:**
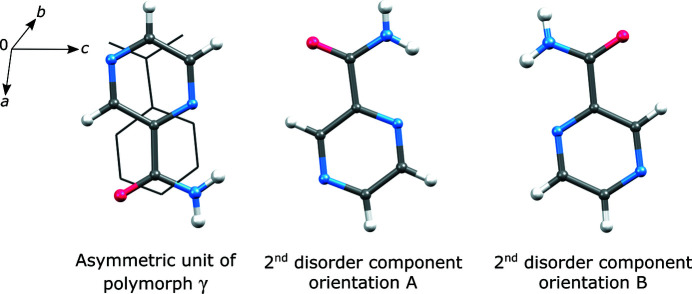
Asymmetric unit of the pyrazinamide γ polymorph showing the backbone of the minor disorder component and two possible orientations of the molecule that may occupy the disordered site.

The γ polymorph is statically disordered. This has been observed also in previous studies (Wahlberg *et al.*, 2014 ▸). Evident electron density maxima showing presence of a second disorder component are present in the Fourier difference maps. In Fig. 5 ▸ the asymmetric unit is depicted showing the major disorder component along with the backbone of the pyrazinamide molecule related to the minor disorder component. Two orientations of the minor site are possible.

Determining which of the orientations occupy the minor disordered site is not straightforward. Furthermore, both presented orientations might be present resulting in three disordered components in total. We did refinements with either the **A** or **B** disorder components present, and found that the *R*_1_ value was more than 1% lower when component **A** was present. In both cases, the disorder component ratio was refined to approximately 85:15. It is consistent with a structure model reported earlier, where the disorder ratio is 87:13 (Cherukuvada *et al.*, 2010 ▸). There, the minor disorder component is also considered in orientation **A**.

**Figure 6:**
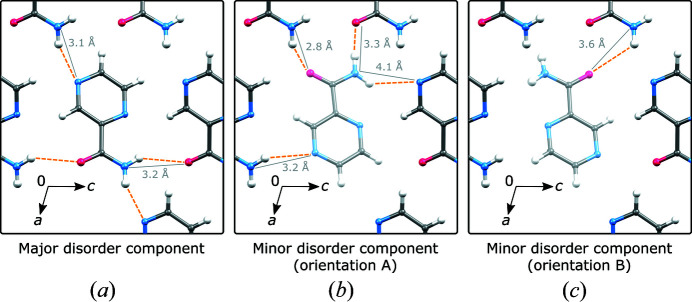
Local structures of (*a*) solely the major disorder component; (*b*) the major disorder component and one molecule of the minor component – orientation **A**; (*c*) the major disorder component and one molecule of the minor component – orientation **B**. *D*⋯*A* distances of possible hydrogen bonds are shown as solid lines, while dashed lines indicate *D*⋯H.

Analysis of hydrogen bonding also hints that a pyrazinamide molecule with orientation **A** would result in an energetically more favorable state. In Fig. 6 ▸(*a*), a structural motif as formed by the major component is depicted also indicating the hydrogen bonding. There are four (two unique) hydrogen bonds associated with each molecule. Expansion of the hydrogen bond network results in a layer. If one molecule is replaced with the minor disorder component in the **A** orientation, then four other hydrogen bonds are possible [see Fig. 6(*b*)]. However, if a local structure with a minor disorder component in its **B** orientation is considered, then only one rather long and thus inefficient hydrogen bond is formed with the molecules of the major disorder component [see Fig. 6 ▸(*c*)].

### 3.3. Lattice and total electronic energies

The lattice energies for pyrazinamide polymorphs were already calculated using various different methods (Wahlberg *et al.*, 2014 ▸). It was found that differences in lattice energies are extremely small and the stability order of the pyrazinamide polymorphs can be reverted by applying a slightly different calculation scheme (*e.g.* by changing a basis set or by small changes of a unit cell).

Herein we conducted once again total and lattice energy calculations at different levels of theory and this time with the D3 dispersion correction. The lowest electronic energy we obtained for the β form, whereas the highest for the α form (see Table 2 ▸). As the high total electronic energy for the α form is in a good agreement with experimental results, such low energy calculated for the β form is surprising – according to the experimental results the most stable form at low temperature should be the δ form. Wahlberg *et al.* (2014 ▸) managed to calculate energies which reproduce experimental stability order by means of theoretical computations with fixed unit-cell parameters at 122 K, with large modified VZP basis set and Grimme dispersion correction. In our case, although we applied a TZVP basis set and D3 dispersion correction, the β form exhibits lower total energy than the δ form.

Regarding the γ form of pyrazinamide, in Table 2 ▸ we present results of calculations which we obtained for the main component, *i.e.* without disorder. In such a case, the γ polymorph have almost equal energy to the δ form. In the real crystal structure, we do observe disorder and thereby the lattice/total electronic energy for the disordered structure is slightly higher than for the hypothetical non-disordered crystal. This destabilization is recompensed at higher temperatures by contributions from the configurational entropy.

In the work of Wahlberg *et al.* (2014 ▸), a 2 × 1 × 1 supercell with *P*1 symmetry was constructed, and one out of eight molecules were flipped to reflect the disorder observed experimentally. In the present case, we have constructed a somewhat larger supercell (2 × 3 × 2) to ensure that the flipped molecules are isolated from each other, in this supercell one out of 24 molecules was flipped. The electronic energy calculated in that manner (B3LYPD3, cell fixed and coordinates optimized) is 0.35 kJ mol^−1^ higher than the energy calculated for pure γ main disorder component (as in Table 2 ▸). Occupancy ratio is 85:15, this would correspond to flipping four molecules out of 24. Considering this occupancy ratio, and with the assumption that the flipped molecules do not interact, we may estimate the electronic energy for the disordered structure to be 4 × 0.35 kJ mol^−1^ higher than for major γ component. It means that the disordered structure is energetically less stable by 1.4 kJ mol^−1^ than the non-disordered γ structure (major component only). When a 1.4 kJ mol^−1^ value is added to the energy obtained for the non-disordered γ structure (from Table 2 ▸, using B3LYPD3 and fixed cell at 10 K, the γ form is −0.9 kJ mol^−1^ more stable than δ) we see that the γ form is becoming less stable than δ by 0.5 kJ mol^−1^ at 0 K.

### 3.4. Entropy, zero-point energy and contribution to enthalpy from lattice vibrations

**Figure 7:**
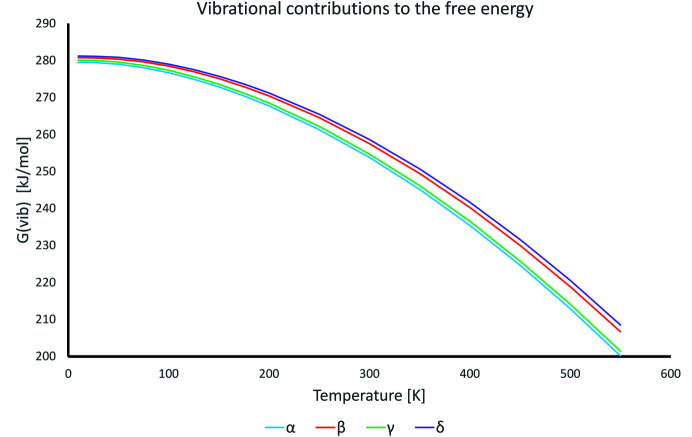
Contributions to the free energy from vibrations (Hvib +ZPE-TS) for four polymorphic forms as a function of temperature.

The entropy and the contribution to enthalpy from lattice vibrations were calculated from frequencies obtained after NoMoRe refinement. Those contributions are temperature dependent and together with ZPE are presented in Fig. 7 ▸. As the vibrational contributions to the free energy are positive, a lower value indicates that the structure is more stabilized by the lattice vibrations. From Fig. 7 ▸ we can immediately observe that with the increase in temperature two forms, α and γ, are starting to have significantly lower energies, stabilized by entropy. At room temperature the energy difference between polymorphic forms is almost 4 kJ mol^−1^ (between δ and α or between γ and β). Such energy differences can easily overcome differences in electronic/lattice energies between polymorphic forms.

In principle, these vibrational contributions can be calculated directly from frequencies obtained from periodic DFT calculations at the Γ point (this is our starting point for NoMoRe). Even with such simple model it appears that the α form is stabilized by entropy, but within this model γ does not exhibit higher vibrational entropy than β (see supporting information). There might be a few explanations for that, and the most obvious one is that with this model we do not consider the change of frequencies due to phonon dispersion. Consequently, we do not consider acoustic modes and some low-frequency modes, which are heavily affected by phonon dispersion and thus might be inaccurately modeled.

Apart from the entropic contributions from vibrations, the configurational entropy, which arises from static disorder, contributes to the γ form free energy. This contribution equals 3.5 J mol^−1^ K^−1^, which at room temperature will lower the free energy by 1.1 kJ mol^−1^, and at 392 K – the temperature of the experimentally estimated phase transition from the α to γ form – around 1.6 kJ mol^−1^.

### 3.5. G-T diagrams

**Figure 8:**
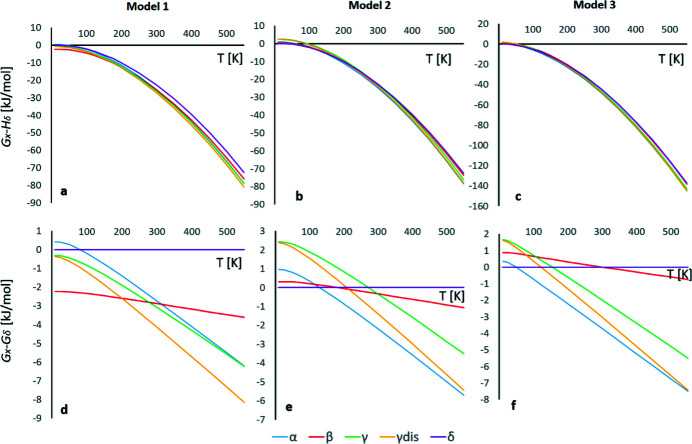
The variation of free energies as a function of temperature. The three models are described in the text. The upper part of the figure shows the total *G*–*T* curves, whereas the lower part shows the differences in free energies with respect to the δ form. The δ form therefore becomes a horizontal line at *G* = 0. For the γdis model we are including the entropy arising from disorder in the free energy calculation for γ.

Based on the crystal energies and the vibrational contributions to entropy and enthalpy derived from the normal-mode refinements, and by considering the consequences of disorder in the γ form for both enthalpy and entropy, it is possible to calculate the free energies as a function of temperature for all four polymorphs (see Fig. 8 ▸). As evident from our calculated energies, it is not straightforward to assess the order of stability at 0 K for the pyrazinamide polymorphs. We therefore make three different curves with different offsets on the abscissa, corresponding to different estimates of the 0 K enthalpies. In terms of the vibrational contribution to the entropy, these models are identical. The three models are presented in the three columns of Fig. 8 ▸. Model 1: periodic DFT (B3LYP, fixed cell, TZP, D3) calculations including destabilization of the γ form due to disorder. Model 2: periodic DFT results from Wahlberg *et al.* (2014 ▸) (Approach III, model B3LYP-D/VTZ-mod in that work). Model 3: average values of experimentally observed enthalpy differences, as observed from DSC measurements (Cherukuvada *et al.*, 2010 ▸). In this model, we assume that the observed enthalpy differences are temperature independent. For this reason, the vibrational enthalpy calculated from the lattice dynamical model was not included.

Comparison of Gibbs free energies obtained from the different models reveals the importance of accurate total electronic and lattice energy calculations (Fig. 8 ▸). Due to those differences, we do observe that free energies obtained from the two theoretical models differ, especially at low temperatures.

### 3.6. Discussion

The vibrational contributions to the free energy, as derived from the NoMoRe procedure, shows that the γ and α forms behave similarly, and are stabilized at higher temperatures as compared to the β and δ forms (see Fig. 7 ▸).

Whereas the vibrational stabilization of α and γ are similar in size, the disorder in the γ form contributes in two ways to form an enantiotropic relationship between these two polymorphs: first, the disorder decreases the packing energy, *i.e.* the γ form is enthalpically destabilized by the disorder. At the same time, the disorder adds a residual entropy component to the γ crystal. At higher temperatures, this entropic stabilization effects that the γ form becomes the stable form. Experimentally, this change in stability is observed at 392 K.

Our 10 K structures confirm the disorder found in the γ form at higher temperatures and at the same time rule out any major disorder in any other forms. These accurate structures, being close to the ‘0 K’ ansatz used in most *ab* *initio* calculations, provide a good benchmark for further optimization of theoretical calculations of polymorphism in organic crystal systems.

Our difficulties in assessing the order of stability of the polymorphs at 0 K imply that many different *G*–*T* diagrams can be constructed, giving very different phase transition temperatures, none of which are in full agreement with the experimentally observed. Since the experimental phase transition temperatures may at the same time be influenced by kinetic factors they may be overestimated. The observed phase transition from δ to α is at 298 K, whereas all our models predict a phase transition well below room temperature. Similarly, our models predict the phase transitions of β to γ to be below room temperature. In contrast, the α to γ transition is predicted to be well beyond room temperature, as observed experimentally.

The effect of thermal expansion and anharmonicity is not part of the NoMoRe approach used in this study. One approach to include anharmonicity would be to impose a quasi-harmonic approach, where a range of NoMoRe models are refined against X-ray diffraction data obtained at differing temperatures. For each temperature, an ansatz could be obtained from periodic DFT calculations performed using the unit-cell dimensions obtained experimentally at that temperature.

Although this approach is not unfolded in the present work, we have performed periodic DFT calculations based on the room-temperature structures obtained from the literature. The calculations of frequencies were performed at the γ point of the Brillouin zone – thus, no contribution from acoustic phonons were included. The thermodynamic properties obtained from these purely in-silico models are compared to similar calculations using the 122 K unit cells in Table S2 of the supporting information. As expected, due to thermal expansion, we observe on average a decrease in the vibrational frequencies at room temperature. This leads to an increased stabilization of all polymorphs. Furthermore, we observe that the delta form is slightly more stabilized compared to the other forms. Thus, including anharmonicity into the models might allow the delta form to be the most stable polymorph in a larger temperature window than we obtain in the *G*–*T* diagrams in Fig. 8 ▸.

## 4. Conclusions

In this contribution, we demonstrate that it is possible to obtain a stability order at temperatures above room temperature which agrees with experimental results. This is obtained by combining vibrational contributions to the free energy estimated from frequencies from NoMoRe with electronic energies from periodic DFT calculations. All our models predict that at high temperatures α and γ are stabilized by entropy and are the most stable polymorphic forms. These observations agree with previous investigations. Moreover, we observe that, due to configurational entropy arising from disorder, the γ form becomes more stable than α at elevated temperatures. Although our models can grasp these gross trends of the polymorph stabilities, we are still quite far away from the observed stabilities at room temperature. The work by Cherukuvada *et al.* (2010 ▸) strongly suggests that the δ form is the most stable below room temperature, whereas the α form is the most stable at room temperature, until eventually the γ form is the most stable until the melting point. We believe that a large part of these discrepancies between our models and the experimentally observed events are due to inaccurate assessment of the electronic energies. Even with a large basis set and D3 dispersion correction we are unable to predict the experimentally most plausible β/δ energy rank at 0 K. Of course, as we have discussed above, there is no direct evidence that the β polymorph is less stable than the δ polymorph at 0 K. Accurate electronic energies/lattice energies are crucial for predicting relative stability of polymorphic systems. It is remarkable that although advanced methods of computational chemistry have been developed there are still problems with calculations of electronic energies for such small rigid system as pyrazinamide, and different approaches lead to different stability ordering. For example: For model 2 (see Fig. 8 ▸) the stabilization of the delta form by 2 kJ mol^−1^ at 0 K would have resulted in a stability ordering at room temperature in close agreement with the observations of Cherukuvada *et al.*

Aree and coworkers (Aree *et al.*, 2013 ▸, 2014 ▸; Aree & Bürgi, 2012 ▸) conducted complete and beautiful analysis of stability ordering for glycine polymorphs. They carefully analyzed lattice energies, vibrational zero-point energies, vibrational enthalpies, and entropies and they found out that the most important contributions to the differences in the free energies of the polymorphs of glycine are the lattice energies, on one hand, and the zero-point energies, on the other. The pyrazinamide polymorphs are different – beside lattice energies, the entropy contributions to free energy differences are crucial for estimating stabilities and phase transition temperatures. Furthermore, it is important to consider not only the vibrational entropies, but also contributions from configurational entropy, which are arising from disorder. Here, we estimated those contributions from occupancies; in future work we will present some more advanced models for configurational entropy estimation for the γ pyrazinamide polymorph.

## Supplementary Material

Crystal structure: contains datablock(s) alpha10K, alpha122K, beta10K, beta122K, gamma10K, gamma122K, delta10K, delta122K. DOI: 10.1107/S2052520622004577/so5076sup1.cif

Crystal energy frameworks, refinement indicators of the NoMoRe refinement, figures of thermal ellipsoids after NoMoRe refinement and contributions to free energy calculations as calculated from periodic DFT calculations. DOI: 10.1107/S2052520622004577/so5076sup2.pdf

CCDC references: 2122633, 2122634, 2122635, 2122636, 2122637, 2122638, 2122639, 2122640

## Figures and Tables

**Table 1:** Crystallographic data of the structure refinements

Phase	α		β		γ		δ	
*T* (K)	10	122	10	122	10	122	10	122
Formula	C_5_H_5_N_3_O							
Formula weight (g mol^−1^)	123.11							
Crystal system	Monoclinic	Monoclinic	Monoclinic	Monoclinic	Monoclinic	Monoclinic	Triclinic	Triclinic
Space group	*P*2_1_/*n*	*P*2_1_/*n*	*P*2_1_/*c*	*P*2_1_/*c*	*Pc*	*Pc*	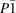	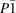
*a* (Å)	3.5885 (9)	3.6330 (10)	14.3433 (9)	14.3280 (3)	7.1818 (7)	7.1730 (17)	5.1026 (6)	5.1280 (14)
*b* (Å)	6.7605 (17)	6.753 (2)	3.6086 (2)	3.6330 (13)	3.6295 (4)	3.6500 (13)	5.7083 (7)	5.7080 (12)
*c* (Å)	22.434 (6)	22.546 (5)	10.6023 (7)	10.6280 (17)	10.6533 (11)	10.686 (2)	9.8487 (12)	9.857 (4)
α (°)	90	90	90	90	90	90	97.526 (4)	97.45 (3)
β (°)	92.748 (8)	92.39 (2)	100.9820 (10)	101.169 (7)	106.3770 (10)	106.487 (17)	98.499 (3)	98.04 (3)
γ (°)	90	90	90	90	90	90	106.564 (4)	106.55 (2)
*V* (Å^3^)	543.6 (2)	552.7 (3)	538.72 (6)	542.7 (2)	266.43 (5)	268.27 (13)	267.37 (6)	269.45 (15)
*Z*, *Z*′	4, 1	4, 1	4, 1	4, 1	2, 1	2, 1	2, 1	2, 1
*F*(000)	256	256	256	256	128	128	128	128
*D*_x_ (g cm^−3^)	1.5042	1.4797	1.518	1.5067	1.5347	1.5241	1.5293	1.5175
μ (mm^−1^)	0.112	0.11	0.113	0.112	0.114	0.113	0.114	0.113
No. of measured reflections	9779	49596	4273	47668	4051	17605	3482	15213
[sin(θ)/λ]_max_ (Å^−1^)	0.831	1.078	0.763	1.078	0.777	1.145	0.835	1.128
No. of unique reflections	2486	5797	1853	5683	1941	5658	2278	4988
No. of observed reflections	1669	3372	1620	3664	1797	3415	1557	4005
Observed condition	*I* > 3σ(*I*)							
*R*_int_	0.048	0.0582	0.0111	0.0392	0.0183	0.0418	0.0258	0.0743
Refinement method	Full-matrix least-squares on *F*							
No. of parameters	82	82	82	82	87	87	37	37
*R*_1_ (obs.)	0.0432	0.0368	0.0333	0.0370	0.0370	0.0474	0.0517	0.0397
*wR* (all)	0.0502	0.0477	0.0467	0.0567	0.0423	0.0545	0.0603	0.0647
GoF (all)	1.72	1.97	2.69	2.73	1.98	1.81	1.89	2.60
H-atom treatment	H-atom parameters constrained							
Weighting scheme	*w* = 1/[σ^2^(*F*)+0.0001*F* ^2^]							
Δ_max_, Δ_min_ (Å^−3^)	0.70, −0.37	0.67, −0.28	0.53, −0.24	0.61, −0.28	0.36, −0.25	0.54, −0.30	0.75, −0.45	0.50, −0.27

**Table 2:** Differences in total electronic energies (kJ mol^−1^) relative to the δ form A negative sign implies that the structure is more stable than δ. Here, the calculations for the γ polymorphic form were conducted for the main disorder component. Level of theory: TZVP (Schäfer *et al.*, 1994 ▸), D3 dispersion correction. Cell (10 K) implies that the cell constants are constrained to the experimentally determined cell at 10 K.

DFT method	Geometry	α	β	γ	δ
B3LYPD3	Cell and coordinates optimised	2.4	−1.9	−1.0	0
PBE0D3	Cell and coordinates optimised	1.8	−1.8	−0.4	0
B3LYPD3	Cell (10 K), coordinates optimised	2.1	−1.8	−0.9	0
PBE0D3	Cell (10 K), coordinates optimised	2.0	−1.4	0.0	0
B3LYPD3	Cell (123 K), coordinates optimised	2.6	−1.7	−0.7	0
