# Supplementary material for: Dynamics and disorder: on the stability of pyrazinamide polymorphs
Source: Acta Crystallogr B Struct Sci Cryst Eng Mater. 2022 Jun 1;78(Pt 3):416–24. doi: 10.1107/S2052520622004577 (PMC9254588; doi:10.1107/S2052520622004577)
Supplement: Supplementary file 2 [file b-78-00416-sup2.pdf]

STRUCTURAL SCIENCE
CRYSTAL ENGINEERING
MATERIALS

Volume 78 (2022)

Supporting information for article:

Dynamics and disorder: on the stability of pyrazinamide polymorphs

Anna Agnieszka Hoser, Toms Rekis and Anders Østergaard Madsen

Figure S1. Contributions of Coulomb and dispersion energies for pyrazinamide polymorphs.

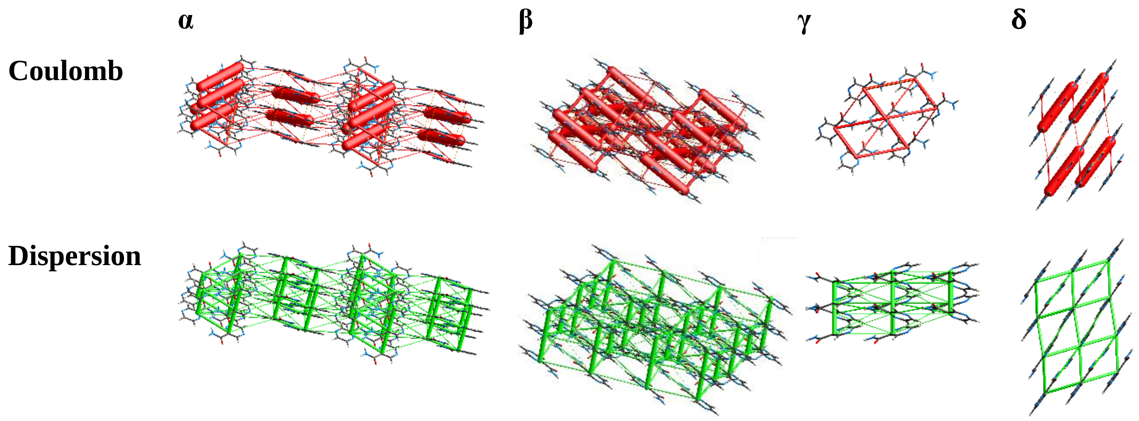

Fig S2. wR2 and ADPs after NoMoRe refinement against 120 K data, 6 frequencies refined. Ellipsoids are shown at the 90 % probability level. Hydrogen atoms are not shown.

	**α**	**β**	**γ**	**δ**
**wR**	0.14	0.12	0.15	0.19

ADPs

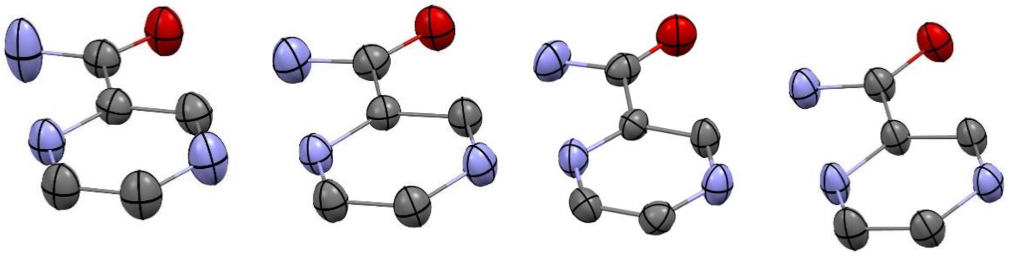

Table S2. Vibrational contributions to total energy (kJ/mol) directly from periodic *ab-initio* calculations B3LYP, pVTZ, ZPE- zero point energy, Hvib-vibrational contributions to enthalpy, S- entropy, Z- number of molecules in the unit cell. Top value – frequencies calculated at unit cell from 120 K, bottom – frequencies calculated at unit cell from room temperature. Thermodynamic values are calculated at 298 K.

	α	β	γ	δ
	1116.2	1121.0	559.6	561.6
**ZPE**	*1115.1*	*1120.3*	*559.0*	*560.2*
	82.2	81.8	38.2	36.8
**Hvib**	*82.5*	*82.3*	*38.4*	*37.3*
	172.3	163.3	74.0	68.5
**TS**	*176.1*	*166.0*	*75.1*	*71.1*
	1026.0	1039.6	523.7	529.8
**Hvib+ZPE-S**	*1021.5*	*1036.6*	*522.3*	*526.4*
	256.5	259.9	261.9	264.9
**(Hvib+ZPE-S)/Z**	*255.4*	*259.1*	*261.1*	*263.2*

Table S3. Contributions to the free energy from vibrations (Hvib +ZPE-TS) for four polymorphic forms as a function of temperature obtained after NoMoRe. Temperature is in Kelvin, energies in kJ/mol.

Temperature	Alpha	Beta	Gamma	Delta
**10**	280	281	280	281
**25**	279	281	280	281
**50**	279	280	280	281
**75**	278	280	279	280
**100**	277	278	277	279
**125**	275	277	276	278
**150**	273	275	274	276
**175**	270	273	271	274
**200**	268	270	268	271
**250**	261	265	262	265
**300**	254	258	255	259
**350**	245	249	246	251
**400**	235	240	237	242
**450**	225	230	226	232
**500**	213	219	214	221
**550**	200	207	201	209

Table S4. Comparison of vibrational contributions to the free energy calculated from frequencies after NoMoRe and purely from DFT calculations at 298K. All values in kJ/mol.

	**α**	**β**	**γ**	**δ**
DFT (CRYSTAL17)	256,5	259,9	261,9	264,9
NoMoRe	254	258	255	259

Table S5. Differences of enthalpies, which were used for G-T diagrams construction (kJ/mol)

	α	β	γ	δ
**Model 1:**				
periodic DFT (B3LYP, fixed cell, TZP, D3)	2.12	-1.79	0.53	0
**Model 2:**				
periodic DFT results from Wahlberg[1] et al., (model B3LYP-D/VTZ-mod)	2.65	0.76	3.57	0
**Model 3:**				
average values of experimentally observed enthalpy differences, as observed from DSC by Cherukuvada[2] et al.	0.36	0.88	1.66	0

[1]*Wahlberg, N., Ciochoń, P., Petriĉek, V. & Madsen, A. Ø. (2014). Crystal Growth & Design* ***14****, 381*
[2]*Cherukuvada, S., Thakuria, R. & Nangia, A. (2010). Crystal Growth & Design* ***10****, 3931*
